# Development of a Chemiluminescence Immunoassay for Quantification of 25-Hydroxyvitamin D in Human Serum

**DOI:** 10.1155/2020/9039270

**Published:** 2020-08-01

**Authors:** Shuang Han, Wuxian Qiu, Junlan Zhang, Zhonghu Bai, Xiao Tong

**Affiliations:** ^1^School of Biotechnology, Jiangnan University, Wuxi, China; ^2^National Engineering Laboratory for Cereal Fermentation Technology, Jiangnan University, Wuxi, China; ^3^Affiliated Hospital of Jiangnan University, Department of Pediatrics, Wuxi, China; ^4^The Key Laboratory of Carbohydrate Chemistry and Biotechnology, Ministry of Education, School of Biotechnology, Jiangnan University, Wuxi 214122, China

## Abstract

In this study, a chemiluminescence immunoassay (CLIA) for human serum 25-hydroxyvitamin D (25(OH)D) was established by a competition model. In serum, more than 99% of total circulating 25(OH)D binds to protein and less than 1% of 25(OH)D is in free form (Jassil et al., 2017). Before measuring concentration of 25(OH)D in serum, a releasing procedure should be conducted. A new reagent is used to release binding 25(OH)D to free form. Streptavidin (SA) was labeled to magnetic beads by a 1-ethyl-3-(3-dimethylaminopropyl) carbodiimide/N-hydroxysuccinimide (EDC/NHS) method. Biotinylated VD was used as a competitor of 25(OH)D in samples. Anti-VD antibody (aby) was labeled to horseradish peroxidase (HRP) by EDC to react with 25(OH)D and biotinylated-VD molecules. The pretreated samples or standards were added into the reaction tube with biotin-VD and anti-VD aby-HRP, free 25(OH)D in the sample competes with biotinylated VD for binding to anti-VD aby-HRP, an SA-labeled magnetic particle is added to isolate the signal-generating complex, and the signal is inversely proportional to the 25(OH)D concentration in the sample. The method established shows good thermostability and performance. The limitation of detection (LoD) is 1.43 ng/mL. The intra-assay coefficient of variation (CV) is 3.66%–6.56%, the interassay CV is 4.19%–7.01%, and the recovery rate is 93.22%–107.99%. Cross-reactivity (CR) was remarkably low with vitamin D2, vitamin D3, 1, 25-dihydroxyvitamin D3, and 1, 25-dihydroxyvitamin D2. At the same time, the cross-reaction values with 25-hydroxyvitamin D2 and 25-hydroxyvitamin D3 were 97% and 100%, respectively. The developed method shows good correlation with the total VD product from Roche and DiaSorin. 1096 clinical patient samples were measured with developed reagent kit in this study. 7 types of disease were involved, and the concentration of 25(OH)D is less than 30 ng/mL in 94.98% of patients.

## 1. Introduction

Vitamin D (VD), also known as sunshine vitamin, plays an important role in bone metabolism [1–3]. Vitamin D deficiency can cause growth retardation and skeletal deficiency in infant and children [4, 5], osteopenia, and osteoporosis usually happen in adult who have low-level vitamin D in circulation [6]. Vitamin D deficiency also has a possible role in chronic diseases, such as cancer [7], autoimmune diseases [8, 9], osteoarthritis [10], diabetes [11], and cardiovascular disease [12]. Therefore, detection of vitamin D concentration is a quite vital requirement of clinical diagnostics. 25(OH)D is the most widely used indicator of vitamin D status in either serum or plasma [13, 14]. There are methods on the market for the analysis of 25(OH)D. A radiological immunoassay was developed by SchiolerV in 1988 [15], which is time consuming and harmful to environment and operator's health. Several automated immunoassays were developed too, such as Liaison® Total Vitamin D, the IDS-iSYS 25-Hydroxy Vitamin D, the ARCHITECT 25-OH Vitamin D, and the ADVIA Centaur® Vitamin D Total, and none of these immunoassays gave results equivalent to the liquid chromatography-tandem mass spectrometry (LC-MS/MS) method [16]. High-performance liquid chromatography spectrometry was developed for 25(OH)D detection [17], but this method is quite expensive and requires special training for the operator. In contrast, CLIA is a simple, sensitive, and cheap method for the high-throughput quantification of analyses in samples. In this study, a direct competitive immunoassay was established on the CLIA platform.

## 2. Materials and Methods

### 2.1. Reagents and Materials

Dynabeads MyOne™ carboxylic acid beads, EZLink™ Sulfo-NHS-LC-Biotinylation Kit, succinimidyl 4-(N-maleimidomethyl)cyclohexane-1-carboxylate (SMCC), and 4′-hydroxyazobenzene-2-carboxylic acid (HABA) solution are obtained from Thermo Fisher; perfluorohexanoate(PFHxA), methanol, EDC, and NHS are purchased from Sigma; an AKTA purifier is purchased from GE healthcare; biotinylated vitamin D (BVD) is obtained from DIASource; HRP is purchased from BBI solutions; streptavidin is purchased from NeuroPeptide from China; a microscope is purchased from Olympus; an automicroplate chemiluminescent analyzer is supplied by Baiming Biotechnology from China; and an automagnetic beads chemiluminescent analyzer is supplied by Zecheng Biotechnology from China. Mice used for antibody production are obtained from Jiangnan University; VD is purchased from Conju-Probe.

### 2.2. Antibody Immunization and Purification

#### 2.2.1. Antibody Immunization

25-hydroxyvitamin D was conjugated to bovine serum albumin (BSA) by a SMCC crosslinker before immunization. Three female BALB/c mice were used as hosts, and each mouse weighed 20g and was 4 weeks old. Hosts were immunized 3 times with 50 ug VD-BSA in complete Freund's adjuvant mixture, and the immunization interval was 3 weeks, at a last boost with 100 ug VD-BSA in incomplete Freund's adjuvant. Splenocytes were harvested from immunized mice in one week, then fusion with SP2/0 cells.

#### 2.2.2. Antibody Screening and Purification

ELISA assay was employed to screen target anti-VD antibody. Biotinylated 25(OH)D was used as a probe. HRP-conjugated anti-mouse antibody was used to generate signals. 96-well plates were coated with streptavidin (SA) as solid phase. One primary screening from the supernatant of splenocyte-SP2/0 fusion cells was performed. 3 rounds of subcloning were conducted to obtain hybridoma-secreting anti-VD monoclonal antibody. The anti-VD monoclonal antibodies were further validated as raw material to build 25(OH)D detection reagent kit.

### 2.3. Reagent Component Preparation

#### 2.3.1. Streptavidin-Coated Magnetic Bead Preparation [18]

Dynabeads MyOne was washed twice with 25 mM 2-morpholinoethanesulfonic acid (MES) buffer, pH 6.0, to remove storage buffer of the particle. EDC and NHS solutions were added to the Dynabeads to active the binding group on particle surface, suspended the particle and well mixed all compounds. The mixture was incubated with gently tilt rotation at room temperature for 30 minutes. The beads were washed two more times to remove the supernatant. The required amount of streptavidin was added into preactivated magnetic beads and incubated for another 30 minutes at room temperature with gently tilt rotation. At last, the particle was washed twice again and then suspended to storage concentration with PBS buffer containing 0.5% BSA, 0.05% polysorbate-20, and 0.02% sodium azide.

#### 2.3.2. Characterization of SA-Coated Magnetic Particle

The biotin-streptavidin system is used to test quality of the in-house prepared magnetic particle. Two sets of reaction tubes are prepared, 30 *μ*L in-house prepared SA-coated magnetic particle is added, and 50 *μ*L biotinylated HRP (50 ng/mL) is added to one set and 50 *μ*L HRP (50 ng/mL) is added to the other set. Both sets were incubated for 30 min at 37°C, and then the particles were washed 3 times with washing buffer. At last, 100 *μ*L substrate reagent is added to generate signals. The RLUs are 710,325 with biotinylated HRP and 352 with HRP. This result indicated that the in-house prepared magnetic particle is ready to use (see Supplementary Table 1).

#### 2.3.3. Antibody Conjugates with HRP

Different weights of anti-VD antibody and EDC are dissolved in separated 0.05 M sodium bicarbonate solutions. Mix them after the solute dissolved completely. HRP is dissolved in 0.1 Mphosphate buffer, pH 7.2. Then, the mixed solution is added into the HRP solution and the pH is lowered to 5.8 with diluted hydrochloric acid. It is incubated for 5 hours at room temperature. Finally, the impurities are removed with a desalination column.

#### 2.3.4. Characterization of Anti-VD Antibody-HRP Conjugates

The anti-VD antibody-HRP conjugate is prepared in different antibody/HRP ratios. Biotin-VD-SA magnetic particle coupling is used as a probe to test quality of the anti-VD antibody-HRP conjugate. Several sets of reaction tubes are prepared, 30 *μ*L biotin-VD-SA magnetic particle coupling is added, and 50 *μ*L different anti-VD antibody-HRP is added with HRP as control into reaction tubes, then incubated for 30 min at 37°C; the particle is washed 5 times with washing buffer. Then, 100 *μ*L substrate reagent is added. The results indicate that anti-VD antibody-HRP conjugates are ready to use (see Supplementary Figure 1).

### 2.4. Reaction Condition Optimization

In serum, more than 99% of total circulating VD binds to protein and less than 1% of VD is in free form. A VD-releasing process should be involved before measuring the concentration of total VD in serum. Alkaline condition and DTT are used by Roche. In this study, a new reagent has been developed to releasing VD which is more efficiency and time-saving; PFHxA and methanol were used as key material in a PH 7.5 buffer. The essential reagents required for this assay include biotin-VD, anti-VD antibody-HRP conjugates, and VD. Upon mixing the anti-VD antibody-HRP conjugates with a serum pretreated containing free VD, a reaction results between the VD and anti-VD antibody. Then, the magnetic particle with binding biotinylated VD was added, competed binding to the remnant anti-VD antibody-HRP conjugate. The RLUs generated are inversely proportional to the VD concentration. By utilizing several different serum references of known VD concentration, a concentration response curve can be generated, and the VD concentration of an unknown can be ascertained. In this study, factors such as VD-releasing procedure, biotin-VD-magnetic particle concentration, anti-VD antibody-HRP type and concentration, and incubation time were optimized.

#### 2.4.1. Method Procedure

We added 30 *μ*L serum and 150 *μ*L VD-releasing reagent into a reacting tube, mixed, and incubated for 18 minutes at 37°C to release VD from binding protein. 50 *μ*L anti-VD antibody-HRP is added into a reacting tube and incubated for 15 minutes at 37°C, then added 30 *μ*L magnetic particle coated with biotinylated VD and incubated for another 5 min at 37°C. After incubation, the magnetic particle was washed three times with washing buffer, and then 200 *μ*L substrate reagent is added to generate signals. A signal reader is used to collect RLUs, and concentration values are calculated if the calibrator curve is available.

#### 2.4.2. Optimization of Biotin-VD and Anti-VD Antibody-HRP Concentration

Different antibody/HRP ratio conjugates present different competing abilities. Signals of standards increase along with the excess molar increase of HRP. The signal (0 ng/mL)/signal (150 ng/mL) value increases first and then remains at the same level, and the largest number is under condition of antibody/HRP ratio at 2 : 1. The results are shown in Supplementary Figure 2. A series concentration of biotinylated VD and anti-VD antibody-HRP was investigated, and the signals and signal (0 ng/mL)/signal (150 ng/mL) value are acceptable when biotin-VD concentration is 30 ng/mL and anti-VD antibody-HRP concentration is 1,000 ng/mL. The results of optimization are shown in Supplementary Table 2.

#### 2.4.3. Optimization of Incubation Time

The biotinylated VD and anti-VD antibody-HRP incubation time is studied, respectively. The procedure remains unchanged described in the method procedure except for the incubation time. For biotinylated VD, signals remain the same in our test time point. For anti-VD antibody-HRP, signals increase as the incubation time is lengthened at first but remain constant while the reaction achieves dynamic equilibrium. Due to time-saving requirement of clinical, we choose 5 min incubation of biotinylated VD and 16 min incubation of anti-VD antibody-HRP as the best incubation condition which achieves high signals with a relative low variable coefficient. The signal results are shown in Supplementary Figure 3 and Table 3.

#### 2.4.4. VD-Releasing Procedure Optimization

PFHxA and methanol were used as key material to release the binding 25(OH)D to free form. PFHxA is used as a main releasing compound while methanol as a cosolvent, with a neutral PH, and the releasing reagent has low effect of following reactions. Theoretically, the more the PFHxA is added, the more efficient the releasing reagent will be; however, according to results with more than 1% PFHxA in the reagent, the final RLU was decreased. Therefore, 1% PFHxA was the chosen concentration. Data are shown in Supplementary Figure 4. The concentration of methanol was optimized, the CV value became better with methanol in the releasing buffer, 5% was the final concentration, and data are shown in Supplementary Figure 5. Finally, the releasing time of 25(OH)D was optimized, data are shown in Supplementary Figure 6. The concentrations of samples hardly increase after 18 mins, so it is chosen as the final releasing time.

### 2.5. Performance Test Method

#### 2.5.1. Limitation of Detection

20 standards with 0 ng/mL analyte are tested, and then MEAN-3SD value is calculated. The corresponding concentration is the limitation of detection.

#### 2.5.2. Precision and Recovery

Three serum samples of different 25(OH)D concentrations were tested and duplicated separately in one experiment and repeated in 20 days, and the intra- and interassay CV are calculated. The concentration of 25(OH)D solutions was added to three serum samples with different analyte levels, and the recovery rate is calculated.

#### 2.5.3. Cross-Reactivity

The specificity of the anti-VD antibody used to selected substances was evaluated by adding the interfering substance to a serum matrix at various concentrations. The cross-reactivity (CR) is defined at the point where the reduction in the signal corresponds to 50% of the signal achieved in the absence of analyte (B/B0 of 50%), as a percentage of the analyte concentration given the same fall in the signal. The CR values were calculated as follows: CR (%) = IC50 of 25(OH)D/IC50 of competitor × 100%.

#### 2.5.4. Accelerated Stability

The whole kit including biotinylated VD, SA-coated magnetic particle, anti-VD antibody-HRP, and VD standards was incubated at 37°C for 7 days, and the signals of standards and samples at different days were compared.

#### 2.5.5. Method Comparison

The established method was compared with two on-market CLIA methods from Roche and DiaSorin. The sample size used to compare with Roche is 244 and ranged from 3 ng/ml to 63.88 ng/ml. 202 serum samples are used to compare with DiaSorin with range from 6.64 ng/ml to 89.00 ng/ml.

## 3. Result

### 3.1. VD-Releasing Reagent Efficiency Test

**Figure 1:**
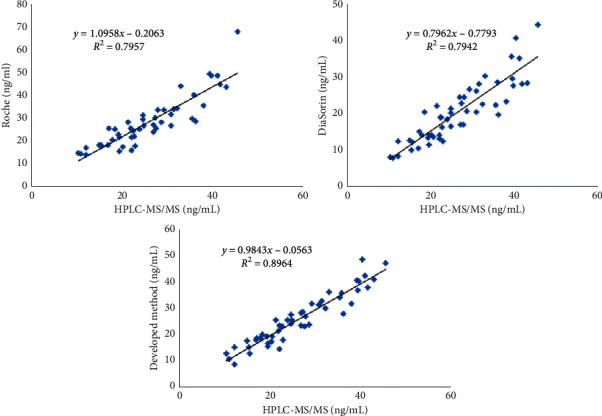
Comparison with UPLC-MS/MS.

Efficiency of the releasing reagent was tested by comparing test results with HPLC-MS/MS concentration. The correlation equation of the developed method with HPLC-MS/MS is *y* = 0.984x−0.056, and correlation coefficient is 0.9468. Results are shown in Figure 1. At the same time, samples were tested on the DiaSorin and Roche platforms side by side. The correlation of the DiaSorin method with HPLC-MS/MS is *y* = 0.796*x* − 0.779, and correlation coefficient is 0.8911. The correlation of the Roche method with HPLC-MS/MS is *y* = 1.095*x* − 0.206, and correlation coefficient is 0.8920.

### 3.2. Method Performance

**Figure 2:**
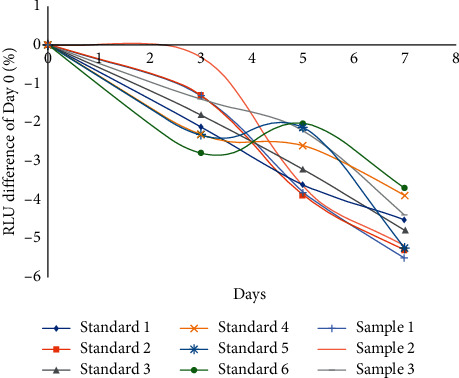
Reagent kit accelerated stability.

The 4-parameter logic function method was used to fit the standard curve of signal with 25(OH)D concentration. The following series concentrations of 25(OH)D were used as a standard curve, 0 ng/mL, 10 ng/mL, 25 ng/mL, 50 ng/mL, 90 ng/mL, and 150 ng/mL. The LoD is 1.43 ng/ml. Precision was tested, intra-assay CV % is 3.66%–6.56%, interassay CV % is 4.19%–7.01%, and the results are shown in Table 1. The recovery rate is 93.22%–107.99%, and the results are shown in Table 2. The developed methods have high selectivity for 25(OH)D. Cross-reaction values were less than 0.1% with vitamin D2, vitamin D3, 1, 25-dihydroxyvitamin D3, and 1, 25-dihydroxyvitamin D2. At the same time, the cross-reaction values with 25-hydroxyvitamin D2 and 25-hydroxyvitamin D3 were 97% and 100%, respectively. Accelerated stability study was performed; the developed reagent showed great stability under stressed temperature. The RLUs changed less than 10% during 7-day periods under 37°C, see the results in Figure 2.

### 3.3. Method Comparison

**Figure 3:**
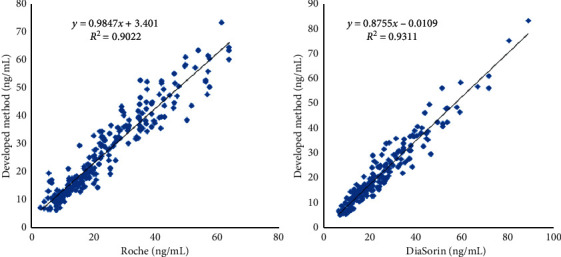
Comparison with on-market products.

In this study, 244 serum samples were measured by both the developed and Roche methods and 202 serum samples were measured by both the developed and DiaSorin methods. The test results were regressed by the least square regression equation, and the correlation coefficient was computed too. Data are shown in Figure 3. Test results show good agreement between the developed method and compared methods, and difference of the test values and mean values indicated slight amounts of bias between developed and compared methods.

### 3.4. Clinical Study

**Figure 4:**
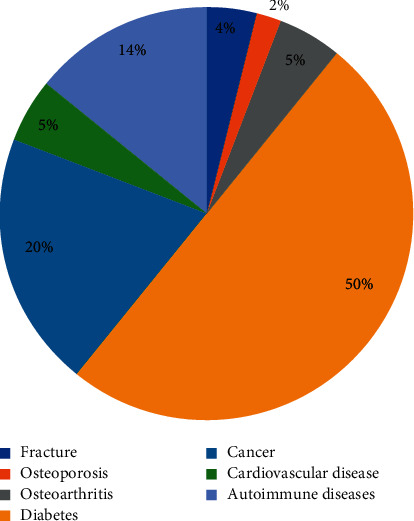
Distribution of clinical samples that 25(OH)D is less than 30 ng/mL.

**Figure 5:**
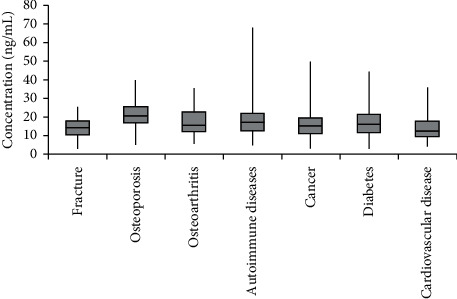
Concentration of 25(OH)D of different patients.

1096 clinical patient samples were measured with developed reagent kit in this study. Samples were treated using the same method we described in Section 2.4.4. 7 types of disease were involved, and the concentration of 25(OH)D is less than 30 ng/mL in 94.98% patients. Data are shown in Table 3. Among patients have low-level 25(OH)D, nearly 50% are related to diabetes, about 20% patients have cancers, and almost 14% got fracture. Data are shown in Figure 4. Patients who have autoimmune disease, cancer, and diabetes have wider range of 25(OH)D level, patients who got fracture, osteoporosis, and osteoarthritis have narrow range of 25(OH)D, and almost all are lower than 30 ng/mL. Data are shown in Figure 5.

## 4. Discussion and Conclusion

According to the previous literature studies, there are many quantification methods for 25(OH)D, such as antibody-based isoluminol derivate direct competitive two-step chemiluminescent system used by DiaSorin; antibody-based acridinium ester competitive assay used by IDS plc, Abbott, and Siemens; 25-OH-vitamin D binding protein based electrochemiluminescence competitive assay used by Roche; and LC-MS/MS-based nonimmunological direct detection method used by PerkinElmer. Due to the differences of the sample pretreatment method and specificity of biomaterial employed in different immunoassays, the agreement of test result is not very good. LC-MS/MS is usually considered as a reference method, but the cost of instruments, consumables, and the low throughput limited its wide application. This study established a chemiluminescence immunoassay for the quantification of 25(OH)D in human serum with good performance and overall stability. The method comparison result shows that this method has a good correlation with the 25(OH)D kit from Roche and DiaSorin which are highly admitted and widely used on the market. Samples results compared with HPLC-MS/MS in releasing reagent optimization section show that this method has a good agreement with the physical detection method which usually be considered as a reference method. In serum, 25(OH)D binds to protein as a complex; pretreatment of the sample should be performed before the test. In this study, a new releasing reagent was employed to maximize the release of 25(OH)D; besides, the PH of the releasing reagent is around 7.5 which has low effect on following reactions. In this study, in-house produced biomaterial such as SA-coated magnetic particle, anti-VD antibody, and anti-VD antibody-HRP conjugate are critical to the reagent performance. Therefore, further studies should be conducted on the antibody-producing procedure and coupling conjugate procedure. Minimization of batch difference of raw material should help to sustain performance of reagent kit. The clinical study with developed kits shows good agreement of the test result and disease, and the reagent kit was fulfilling for the clinical requirement.

## Figures and Tables

**Table 1:** Precision result.

Samples	Intra-assay CV (*n* = 20)			Interassay CV (*n* = 20)		
	Mean (ng/mL)	SD	CV (%)	Mean (ng/mL)	SD	CV (%)
1	9.91	0.65	6.56	9.56	0.67	7.01

2	24.93	1.13	4.53	24.37	1.02	4.19
3	50.52	1.85	3.66	50.18	2.14	4.26

**Table 2:** Analytical recovery.

Samples	VD concentration (ng/mL)			
	Added concentration	Tested	Expected	Recovery (%)
1	0.00	9.76	10.54	107.99
	5.63	15.88	15.48	97.48
	11.25	20.98	19.90	94.85
	22.50	31.18	31.97	102.53
	45.00	58.99	54.99	93.22

2	0	23.53	24.56	104.38
	5.625	29.59	29.21	98.72
	11.25	35.10	34.87	99.34
	22.5	47.20	45.76	96.95
	45	65.29	67.90	104.00

**Table 3:** Distribution of clinical samples.

Conc. of 25(OH)D	Fracture	Osteoporosis	Osteoarthritis	Autoimmune diseases	Cancer	Diabetes	Cardiovascular disease
<30 ng/mL	41	25	55	140	203	524	53
>30 ng/mL	0	4	1	16	6	27	1
Total	41	29	56	156	209	551	54

## Data Availability

The data used to support the findings of this study are available from the corresponding author upon request.

## Supplementary Materials

Supplementary Materials Figure S1: characterization of anti-VD antibody-HRP conjugate (*n* = 3). Figure S2: performance of different antibody/HRP ratio conjugates. Figure S3: optimization of incubation time. Figure S4: optimization of PFHxA concentration. Figure S5: optimization of methanol concentration. Figure S6: optimization of VD-releasing time. Table S1: characterization of the magnetic particle. Table S2: biotin-VD and anti-VD antibody-HRP concentration optimization. Table S3: precision results of optimization incubation time.

## References

[1] Jassil N. K., Sharma A., Bikle D., Wang X. (2017). Vitamin D binding protein and 25-hydroxyvitamin D levels: emerging clinical applications. *Endocrine Practice*.

[2] Anderson P. H. (2017). Vitamin D activity and metabolism in bone. *Current Osteoporosis Reports*.

[3] Lips P., van Schoor N. M. (2011). The effect of vitamin D on bone and osteoporosis. *Best Practice & Research Clinical Endocrinology & Metabolism*.

[4] Hollis B. W., Wagner C. L. (2004). Assessment of dietary vitamin D requirements during pregnancy and lactation. *The American Journal of Clinical Nutrition*.

[5] Hollis B. W., Wagner C. L. (2004). Vitamin D requirements during lactation: high-dose maternal supplementation as therapy to prevent hypovitaminosis D for both the mother and the nursing infant. *The American Journal of Clinical Nutrition*.

[6] Michael F., Holick M. D. (2007). Vitamin D deficiency. *New England Journal of Medicine*.

[7] Mondul A. M., Weinstein S. J., Layne T. M., Albanes D. (2017). Vitamin D and cancer risk and mortality: state of the science, gaps, and challenges. *Epidemiologic Reviews*.

[8] Lee YH., Bae S. C. (2016). Vitamin D level in rheumatoid arthritis and its correlation with the disease activity: a meta-analysis. *Clinical and Experimental Rheumatology*.

[9] Smyk D. S., Orfanidou T., Invernizzi P., Bogdanos D. P., Lenzi M. (2013). Vitamin D in autoimmune liver disease. *Clinics and Research in Hepatology and Gastroenterology*.

[10] Manoy P., Yuktanandana P., Tanavalee A. (2017). Vitamin D supplementation improves quality of life and physical performance in osteoarthritis patients. *Nutrients*.

[11] Wimalawansa S. J. (2018). Associations of vitamin D with insulin resistance, obesity, type 2 diabetes, and metabolic syndrome. *The Journal of Steroid Biochemistry and Molecular Biology*.

[12] Scragg R., Stewart A. W., Waayer D. (2017). Effect of monthly high-dose vitamin D supplementation on cardiovascular disease in the vitamin D assessment study. *JAMA Cardiology*.

[13] Hollis B. W. (2008). Measuring 25-hydroxyvitamin D in a clinical environment: challenges and needs. *The American Journal of Clinical Nutrition*.

[14] holick M. F. (2009). Vitamin D status: measurement, interpretation, and clinical application. *Annals of Epidemiology*.

[15] SchiolerV T.J. (1988). Six direct radioimmunoassay of estradiol evaluated. *Clinical Chemistry*.

[16] Koivula M.-k., Matnlassi N., Paivilaitinen J. R. (2013). Four automated 25-OH total vitamin D immunoassays and commercial liquid chromatography tandem-mass spectrometry in Finnish population. *Clinical Laboratory*.

[17] Plíšek J., Krčmová L. K., Aufartová J. (2013). New approach for the clinical monitoring of 25-hydroxyvitamin D3 and 25-hydroxyvitamin D2 by ultra high performance liquid chromatography with MS/MS based on the standard reference material 972. *Journal of Seperation Science*.

[18] Chen X., Zhou Q., Zhang T. (2017). Development of a sensitive chemiluminescence immunoassay for the quantification of folic acid in human serum. *Journal of Analytical Methods in Chemistry*.

