# Supplementary material for: Development of a Chemiluminescence Immunoassay for Quantification of 25-Hydroxyvitamin D in Human Serum
Source: J Anal Methods Chem. 2020 Aug 1;2020:9039270. doi: 10.1155/2020/9039270 (PMC7416287; doi:10.1155/2020/9039270)
Supplement: Supplementary Materials — Figure S1: characterization of anti-VD antibody-HRP conjugate (n = 3). Figure S2: performance of different antibody/HRP ratio conjugates. Figure S3: optimization of incubation time. Figure S4: optimization of PFHxA concentration. Figure S5: optimization of methanol concentration. Figure S6: optimization of VD-releasing time. Table S1: characterization of the magnetic particle. Table S2: biotin-VD and anti-VD antibody-HRP concentration optimization. Table S3: precision results of optimization incubation time. [file 9039270.f1.doc]

Supplementary material for Development of a Chemiluminescence Immunoassay for Quantification of 25-hydroxyvitamin D in Human Serum

Table 1 Characterization of Magnetic Particle

|  | RLUs | |
| --- | --- | --- |
|  | Test | Control |
| SA-magnetic particle | 710,325 | 352 |

Use biotin-streptavitin system to test quality of in house prepared magnetic particle. Prepare two sets of reaction tube, add 30μL in house prepared SA-coated magnetic particle, and add 50μL biotinylated HRP (50ng/mL) to one set, add 50μL HRP (50ng/mL) to the other set. Both sets were incubated for 30min at 37℃, and then the particles were washed 3 times with washing buffer. At last, add 100μL substrate reagent to generate signals. The RLUs are 710,325 with biotinylated HRP, 352 with HRP. This result indicated that in house prepared magnetic particle is ready to use.


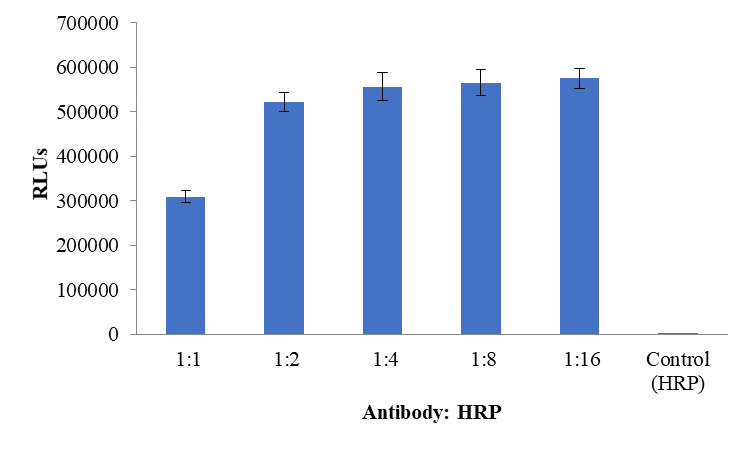


Figure 1 Characterization of Anti-VD antibody-HRP Conjugate (n=3)

Prepare anti-VD antibody-HRP conjugate in different antibody/HRP ratio. Use Biotin-VD-SA magnetic particle coupling as probe to test quality of anti-VD antibody- HRP conjugate. Prepare several sets of reaction tubes, add 30μL Biotin-VD-SA magnetic particle coupling, and add 50μL different anti-VD antibody-HRP with HRP as control into reaction tubes, then incubate for 30min at 37°C, and wash the particle 5 times with washing buffer. Then add 100μL substrate reagent. The results indicate that anti-VD antibody-HRP conjugates are ready to use.

Table 2 Biotin-VD and anti-VD antibody-HRP Concentration Optimization

| Biotin-VD(ug/mL) |  | 0.06 |  |  | 0.03 |  |  | 0.015 |  |
| --- | --- | --- | --- | --- | --- | --- | --- | --- | --- |
| Anti-VD antibody-HRP (ug/mL) | 2 | 1 | 0.5 | 2 | 1 | 0.5 | 2 | 1 | 0.5 |
| Standard 1 (0 ng/mL) | 829077 | 813300 | 374810 | 803570 | 556758 | 172595 | 312341 | 286217 | 82400 |
| Standard 2 (10 ng/mL) | 790664 | 525689 | 177314 | 500115 | 274156 | 93662 | 171971 | 150494 | 41941 |
| Standard 3 (25 ng/mL) | 436673 | 261481 | 96885 | 267598 | 152913 | 57165 | 95406 | 85631 | 26240 |
| Standard 4 (50 ng/mL) | 298517 | 162914 | 63392 | 181122 | 95832 | 35458 | 57039 | 46541 | 12573 |
| Standard 5 (90 ng/mL) | 155716 | 101261 | 45130 | 101261 | 57217 | 18949 | 33227 | 26201 | 19593 |
| Standard6 (150 ng/mL) | 105587 | 63783 | 27835 | 64461 | 33927 | 16892 | 21225 | 19017 | 15822 |

A series concentration of biotinylated VD and anti-VD antibody-HRP were investigated, the signals and Signal (0ng/mL)/Signal (150ng/mL) value are acceptable when biotin-VD concentration is 30 ng/mL and anti-VD antibody-HRP concentration is 1,000 ng/mL.


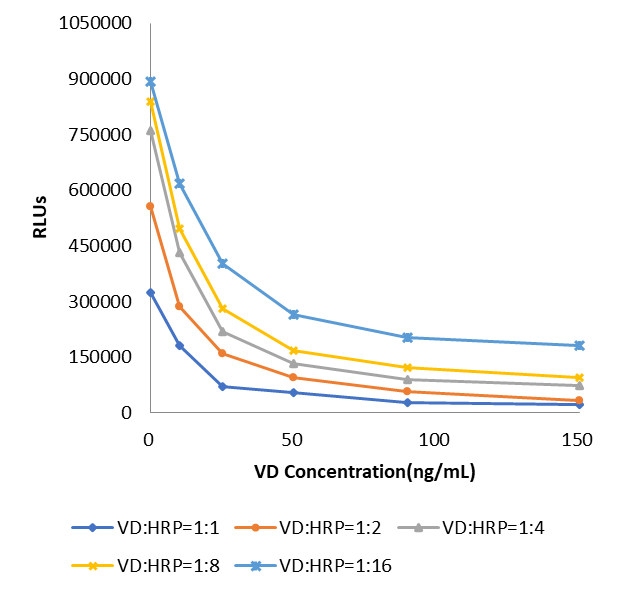


Figure 2 Performance of different antibody/HRP ratio conjugates

Different antibody/HRP ratio conjugates present different competing abilities. Signals of standards increase along with the excess molar increase of HRP. Signal (0ng/ mL)/Signal (150ng/mL) value increase first then remain at the same level, the largest number is under condition that antibody/HRP ratio at 2:1.


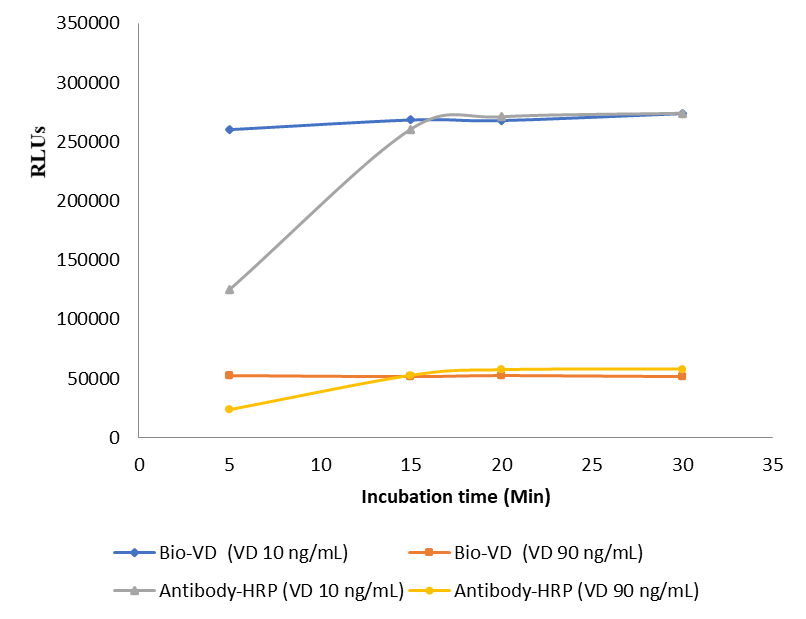


Figure 3 Optimization of incubation time

Table 3 Precision results of optimization incubation time

| Standards | Mean | SD | CV (%) |
| --- | --- | --- | --- |
| 10 ng/mL | 9.982 | 0.22 | 2.20 |
| 90 ng/mL | 87.804 | 1.73 | 1.97 |

The biotinylated VD and anti-VD antibody-HRP incubation time is studied, respectively. The procedure remains unchanged described in method procedure except the incubation time. For biotinylatd VD, signals remain the same in our test time point. For anti-VD antibody-HRP, signals increase as the incubation time is lengthened at first, but remains constant while the reaction achieves dynamic equilibrium. Due to time-saving requirement of clinical, we choose 5min incubation of biotinylated VD and 16 min incubation of anti-VD antibody-HRP as the best incubation condition which achieve high signals with a relative low variable coefficient.


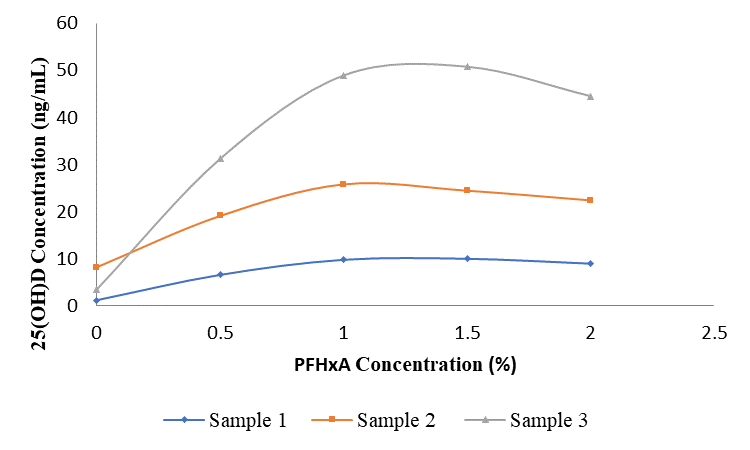


Figure 4 Optimization OF PFHxA Concentration

PFHxA and methanol were used as key material to release the binding 25(OH)D to free form. PFHxA used as main releasing compound while methanol as cosolvent, with a neutral PH, the releasing reagent has low effect of following reactions. Theoretically, the more PFHxA added the more efficient the releasing reagent will be, however, according to results with more than 1% PFHxA in the reagent, the final RLU was decrease. Therefore 1% PFHxA was the chosen concentration.


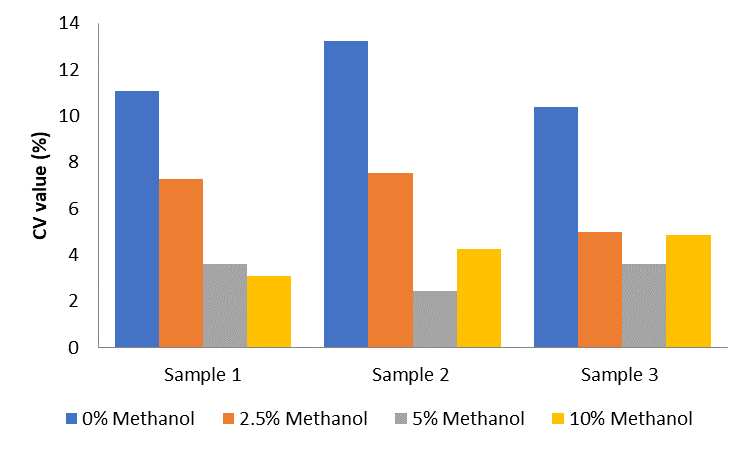


Figure 5 Optimization of methanol concentration

The concentration of methanol was optimized, the CV value became better with methanol in the releasing buffer, 5% was the final concentration


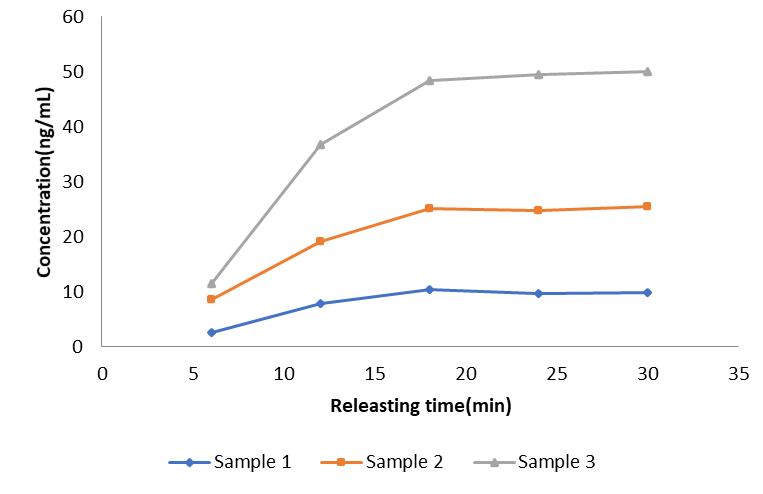


Figure 6 Optimization of VD releasing time

The releasing time of 25(OH)D was optimized. The concentrations of samples hardly increase after18mins, so it is chosen as the final releasing time.
